# Unveiling patterns of peri-lead edema after deep brain stimulation: a retrospective review of clinical and demographic factors

**DOI:** 10.1007/s00234-025-03607-z

**Published:** 2025-04-08

**Authors:** Coplen Johnson, Garret Miller, Shivam Shah, Christopher Stevens, Nicholas Thomas, Jamie Toms, Octavio Arevalo

**Affiliations:** Louisiana State University Health Sciences Center Shreveport, Shreveport, USA

**Keywords:** Deep Brain Stimulation, Peri-Lead Edema, Neurosurgery, Radiology, Neuroradiology, Neurology, Brain

## Abstract

**Objective:**

Postoperative peri-lead edema (PLE) is an increasingly recognized complication of deep brain stimulation (DBS), a therapeutic intervention commonly used for neurological conditions such as Parkinson’s disease (PD), essential tremor (ET), intractable focal epilepsy, and dystonia. In this study, we conducted a retrospective chart review to evaluate the incidence of PLE and explore potential clinical and demographic risk factors.

**Methods:**

A single physician performed DBS electrode placements. To check for complications, postoperative computed tomography (CT) scans were conducted on the day of surgery and approximately 12–15 days afterward. Data on age, gender, complications, edema size, electrode laterality, lead target, lead brand, indication, and use of robotic assistance versus Starfix were collected and analyzed statistically.

**Results:**

133 leads were implanted in 63 patients, with peri-lead edema (PLE) observed in 65 electrodes (48.87%). Minor postoperative complications, such as subarachnoid hemorrhage (SAH) and subdural hematoma (SDH), were noted in some patients. A few cases of severe PLE were recorded, with the most significant volume of edema reaching 85.11 cm³. No statistically significant differences were found between PLE-positive and PLE-negative patients based on age, sex, lead target, indication, or robotic assistance versus Starfix. However, the use of Boston Scientific electrodes was significantly associated with PLE, with a p-value of 0.047. A logistic regression model (*p* = 0.013, R² = 0.219) correctly classified 63.2% of cases, with no significant predictors of PLE, but imaging complications (*p* = 0.057) and electrode brand (*p* = 0.086) approached significance, with Boston Scientific electrodes linked to higher PLE risk compared to Abbott electrodes (*p* = 0.027, OR = 3.729).

**Conclusions:**

PLE appears more prevalent than previously reported and generally presents with delayed onset post-surgery. This retrospective analysis identified the use of Boston Scientific electrodes as a potential risk factor for PLE. Further research, particularly more extensive studies, is necessary to clarify the underlying mechanisms of PLE, improve prevention strategies, and enhance our understanding of this complication.

## Introduction

Deep Brain Stimulation (DBS) has become an increasingly beneficial therapy for patients with a range of neurological disorders, including PD, ET, and dystonia [1]. The main targets for electrode placement are deep parts of the brain, like the ventral intermediate nucleus (VIM), subthalamic nucleus (STN), and globus pallidus internus (GPi) [2]. The electrodes, connected to a pulse generator usually placed under the skin near the clavicle, are surgically implanted in the brain area based on the specific disorder being treated [3]. Although different areas of the brain may be targeted besides the subthalamic nucleus, like the GPi and VIM, all these regions are involved in crucial movement control in the basal ganglia network pathway [1]. The exact mechanism of the impulse effect is still being studied, but stimulation, inhibition, and jamming of neural circuits are some of the proposed mechanisms [4].

**Fig. 1:**
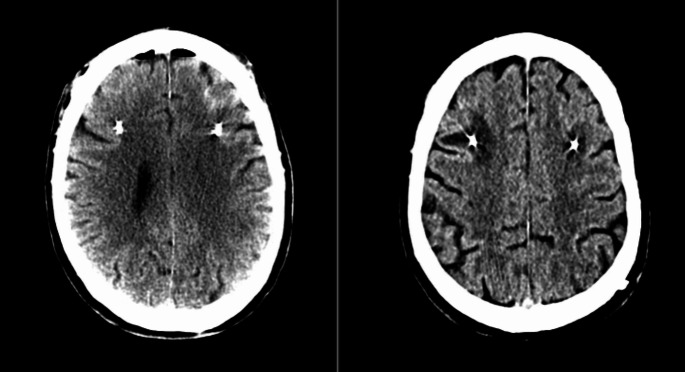
Postoperative same-day CT scan showing no edema (left) compared to a follow-up CT scan weeks later revealing the presence of edema (right)

Despite its success, complications following DBS implantation have been reported. One such concern is the presence of Peri-Lead Edema (PLE), the localized swelling around the implanted DBS electrodes which shows up on CT imaging as hypodensity surrounding the electrode (Fig. 1) [5]. PLE has increasingly become a studied clinical complication of DBS that has yet to be fully understood. Various studies have shown that PLE leads to adverse effects, usually showing altered mental status or neurological function in 3–31.6% of patients [5–7]. The underlying mechanism and presence of PLE in DBS patients and its role in post-surgical complications are still being studied.

Understanding the prevalence and potential risk factors for PLE has several critical implications for patient care. First, patients undergoing DBS should be counseled preoperatively about the possibility of developing PLE, including its delayed onset, to ensure appropriate expectations and compliance with follow-up imaging protocols. Second, the identification of PLE in postoperative imaging could inform tailored management strategies, such as closer neurological monitoring or adjustments to stimulation parameters, to mitigate clinical sequelae associated with severe edema. This retrospective study aims to assess the incidence of PLE and identify potential clinical and demographic risk factors. A key strength of this study lies in its focus on an underexplored area: the direct comparison of implantation techniques (StarFix vs. robotic guidance) and electrode brands (Boston Scientific vs. other manufacturers). By addressing this gap in the existing literature, our study provides novel insights into factors that may contribute to PLE, building upon prior research while offering new avenues for investigation.

## Methods

A retrospective study was conducted to assess the incidence and clinical significance of peri-lead edema (PLE) in 63 patients who underwent deep brain stimulation (DBS) implantation. Patients were included if they received DBS electrodes placed by a single experienced neurosurgeon between March 2022 and April 2024. All patients underwent thorough preoperative evaluations to confirm surgical candidacy. Electrode systems from Abbott, Medtronic, Neuropace, or Boston Scientific were implanted unilaterally or bilaterally using stereotactic techniques, either with the StarFix platform or robotic assistance. Electrode selection was based on device availability at the time of surgery. Importantly, the operating neurosurgeon had extensive experience with DBS implantation, minimizing variability due to surgical technique. Neuropace electrodes were specifically utilized in patients with intractable focal epilepsy, comprising only four electrodes across two patients. All leads used in this study were directional, and microelectrode recordings (MER) were performed in every case under general anesthesia.

### Ethics and funding

The study received approval from the Louisisana State University Health Shreveport Institutional Review Board (STUDY00002577) and was conducted in accordance with the Declaration of Helsinki. Patient consent was not obtained since this was a retrospective analysis in which data was collected via chart review and there is no information revealing patient identity in the manuscript. No funding was needed for this project.

### Data collection

Data was extracted from electronic medical records, including demographics (age, sex), preoperative and postoperative imaging dates, the presence and size of PLE, the number of electrodes, electrode laterality, target site, electrode brand, surgical indication, and robotic assistance versus Starfix.

### Imaging

Preoperative and postoperative CT scans were reviewed by a single board certified neuroradiologist. Postoperative imaging was obtained on the day of surgery and approximately 12–15 days after to monitor for complications. The neuroradiologist evaluated all scans to assess for PLE and any other complications associated with electrode placement. PLE was considered to be a newly developed well circumscribed hypodensity of the brain parenchyma surrounding the lead, easily identifiable using the standard brain window level and width.

### Statistical analysis

Patients with peri-lead edema were compared to those with normal postoperative CT findings using Fisher’s exact, Chi-Square, and T-test, with statistical significance defined as *P* < 0.05. Variables identified in the univariable analyses were included in a binomial logistic regression using a backward stepwise selection method to identify factors associated with PLE. For variables with several categories, the category with the greatest number of patients was considered the reference value for comparison. The best valid model, which classified the highest percentage of participants, was selected based on a combination of the omnibus tests of model coefficients, R² Nagelkerke, and the Hosmer–Lemeshow test. Statistical analyses were performed using SPSS version 29.

## Results

### Patient and electrode characteristics

A total of 133 electrodes were implanted in 63 patients, with peri-lead edema (PLE) observed in 48.87% (*n* = 65) of the electrodes. Minor postoperative complications, such as subarachnoid hemorrhage SAH and SDH, were noted but resolved spontaneously without the need for medical intervention. In a few cases, severe PLE was recorded, with the largest volume of edema measuring 85.11 cm³, indicating a substantial response to electrode implantation in some patients. Furthermore, the occurrence of edema exceeding 20 cc across a wide range of patient demographics indicates that its development is not confined to any specific group and is likely influenced by multiple factors. This observation underscores the need for further investigation in future studies to better understand the underlying contributors.

### Demographics and statistical analysis

The demographic and clinical characteristics between PLE-positive and normal CT groups were analyzed, revealing no significant differences across multiple variables (Table 1). The age of patients in the PLE-positive group (64.58 ± 10.33 years) was similar to that in the normal group (65.40 ± 10.84 years), with a p-value of 0.659, indicating no age-related association with the presence of PLE. It is important to note that the two patients with intractable focal epilepsy were on average 30 years younger than the PD and ET patients. Gender distribution (M/F: 36/29 in PLE-positive vs. 40/28 in normal, *p* = 0.689) and electrode laterality (L/R: 32/33 in PLE-positive vs. 33/35 in normal, *p* = 0.936) were also comparable between the groups.

**Table 1:** Demographics of patients with PLE on CT vs. patients with normal CTa

	PLE (*n* = 65)^b^	Normal (*n* = 68)^c^	*P* Value
Gender (M/F)	36/29	40/28	0.689
Age (in Years)	64.58 ± 10.332	65.40 ± 10.842	0.659
Electrode Laterality (L/R)	32/33	33/35	0.936
Type of Frame (Robot/Starfix)	24/41	28/40	0.615
Presence of Complications	11/65	20/68	0.089
Presence of PD	47.69	47.06	0.942
Presence of ET	50.77	44.12	0.443
Presence of Intractable Focal Epilepsy	0.00	5.88	0.120
Presence of Dystonia	7.69	5.88	0.741

PLE, peri-lead edema; M, male; F, female; L, left; R, right; PD, Parkinson disease; ET, essential tremor; CT, computed tomography

^a^Data is represented as a percent unless otherwise indicated

^b^Number of leads with signs of PLE on CT 12–15 days after implantation

^c^Number of leads with no signs of PLE on CT 12–15 days after implantation

Additionally, there was no significant difference in the rates of complications with complications being more common in patients without PLE (29.4%) than patients with PLE (16.9%, *p* = 0.089).

The presence of PD did not differ significantly between the two groups, with a prevalence of 47.69% in PLE-positive patients and 47.06% in the normal group (*p* = 0.942). ET was slightly more common in the PLE-positive group (50.77%) compared to the normal group (44.12%), though this was not statistically significant (*p* = 0.443). The use of robotic guidance versus the Starfix system did not show a significant association with PLE, with usage rates of 24/41 in PLE-positive and 28/40 in normal patients (*p* = 0.615). Additionally, the presence of intractable focal epilepsy was noted only in the normal group (5.88%, *p* = 0.120), while dystonia prevalence was similar across both groups (7.69% in PLE-positive vs. 5.88% in normal, *p* = 0.741).

### Electrode types

A statistically significant association was found between using Boston Scientific electrodes and PLE (*p* = 0.047). Specifically, 23.08% of PLE-positive patients had Boston Scientific leads compared to only 10.29% in the normal group, suggesting a possible link between electrode type and the development of PLE (Table 2). In contrast, Abbott electrodes were slightly more prevalent in the normal group (82.35%) than in the PLE-positive group (76.92%), although this difference was not statistically significant (*p* = 0.436). Medtronic electrode use was minimal and comparable in both groups (0.00% in PLE-positive vs. 1.47% in normal, *p* = 1.000), and there was no significant association with Neuropace electrodes (0.00% in PLE-positive vs. 5.88% in normal, *p* = 0.120).

**Table 2:** Electrode types used in patients with PLE on CT vs. patients with normal CTa

	PLE (*n* = 65)^b^	Normal (*n* = 68)^c^	*P* Value
Use of Abbott Electrode	76.92	82.35	0.436
Use of Boston Scientific Electrode	23.08	10.29	0.047
Use of Medtronic Electrode	0.00	1.47	1.000
Use of Neuropace Electrode	0.00	5.88	0.120

PLE, peri-lead edema; L, left; R, right; CT, computed tomography

^a^Data is represented as a percent

^b^Number of leads with signs of PLE on CT 12–15 days after implantation

^c^Number of leads with no signs of PLE on CT 12–15 days after implantation

### Electrode placement

The most common electrode placement target was the VIM (Table 3), used in 63.08% of PLE-positive patients and 66.18% of normal patients (*p* = 0.709). Other placements included GPi, with no significant difference between PLE-positive (30.77%) and normal patients (27.94%, *p* = 0.720), and STN, which trended toward significance (*p* = 0.054) with a small number of placements (6.15% in PLE-positive, 0.00% in normal).

**Table 3:** Electrode location in patients with PLE on CT vs. patients with normal CTa

	PLE (*n* = 65)^b^	Normal (*n* = 68)^c^	*P* Value
Electrode Placed at VIM	63.08	66.18	0.709
Electrode Placed at GPi	30.77	27.94	0.720
Electrode Placed at STN	6.15	0.00	0.054
Electrode Placed at Hippocampus	0.00	5.88	0.120

PLE, peri-lead edema; VIM, ventral intermediate nucleus; GPi, globus pallidus internus; STN, subthalamic nucleus; CT, computed tomography

^a^Data is represented as a percent

^b^Number of leads with signs of PLE on CT 12–15 days after implantation

^c^Number of leads with no signs of PLE on CT 12–15 days after implantation

### Factors associated with PLE

The best valid model (*p* = 0.013, R^2^ Nagelkerke = 0.219, Hosmer–Lemeshow test = 0.776) classified 63.2% (*n* = 133) of participants. None of the factors that were studied seemed to have any significant effect in the presence of PLE, however the presence of complications (subarachnoid hemorrhage, encephalomalacia, and subdural hematomas) on CT/MRI (*p* = 0.057, OR = 0.380, 95% CI = 0.141–1.028) and brand of electrode (*p* = 0.086) approached significance. While the overall categorical variable for the brand of electrode was not significant (*p* = 0.086), a pairwise comparison suggested that using Boston Scientific may increase the odds of PLE by 3.729 times compared to Abbott (*p* = 0.027, OR = 3.729, 95% CI = 1.164–11.943) (Table 4). A logistic regression model (*p* = 0.013, R² = 0.219) correctly classified 63.2% of cases, with no significant predictors of PLE, but imaging complications (*p* = 0.057) and electrode brand (*p* = 0.086) approached significance, with Boston Scientific electrodes linked to higher PLE risk compared to Abbott electrodes (*p* = 0.027, OR = 3.729).

**Table 4:** Factors associated with PLE in patients after deep brain stimulation

	β	*P* value	OR (95% confidence interval)
Age at Time of Surgery	-0.030	0.162	0.971 (0.931–1.012)
Presence of Complications on CT/MRI	-0.967	0.057	0.380 (0.141–1.028)
Electrode Laterality (L/R)	-0.030	0.938	0.971 (0.461–2.043)
Brand of Electrode (Reference: Abbott)	-	0.086	-
Boston Scientific vs. Abbott	1.316	0.027	3.729 (1.164–11.943)
Medtronic/Neuropace vs. Abbott	-42.319	0.999	0.000 (0-)
Type of Frame	-0.198	0.639	0.820 (0.358–1.877)
Type of Conditions Found (Reference: PD)	-	0.749	-
Dystonia/Intractable Focal Epilepsy vs. PD	-0.031	0.976	0.969 (0.126–7.459)
ET vs. PD	0.564	0.273	1.758 (0.642–4.818)
Multiple Conditions vs. PD	0.122	0.910	1.130 (0.136–9.378)
Electrode Location (Reference: VIM)	-	0.776	-
GPi vs. VIM	0.383	0.476	1.466 (0.512–4.199)
STN/Hippo vs. VIM	21.416	0.999	1998248456.7 (0-)

PLE, peri-lead edema; M, male; F, female; L, left; R, right; PD, Parkinson disease; ET, essential tremor; CT, computed tomography

## Discussion

This study demonstrates that PLE is a frequent postoperative complication, occurring in nearly half of the electrodes implanted during DBS. The incidence of PLE in our study is notably higher than previously reported rates [5, 8]. Importantly, postoperative imaging via CT was conducted on the day of surgery and again 12–15 days later during the follow-up visit. No signs of PLE were observed immediately after surgery; all PLE-positive cases were identified at the two-week follow-up, reinforcing the consensus that PLE typically presents with delayed onset [5]. Additionally, this is the first study to date that examines the association between specific electrode brands and the occurrence of PLE. Our data showed that the use of Boston Scientific electrodes was associated with a higher incidence of PLE compared to Abbott electrodes, indicating that device-specific factors may contribute to its development. We also found no significant associations between PLE and patient demographics or surgical techniques. For instance, if human error were a factor, one might expect the incidence of PLE to be significantly higher in the StarFix group compared to the robot-assisted group, but this was not the case with our data analysis. STN placements, although not statistically significant, showed a trend toward significance with PLE, suggesting that this target site may predispose patients to developing PLE more than other commonly targeted regions, such as VIM or GPi. The STN may be more vulnerable to edema due to its unique vascular or anatomical properties, or it may provoke a heightened inflammatory response when electrodes are implanted in this region. If confirmed in future studies, this potential site-specific risk could impact surgical planning, particularly for patients requiring DBS in the STN for conditions like Parkinson’s disease [9]. Further research is essential to clarify whether the STN placement trend is clinically significant, as a clearer understanding of target-specific risks could help optimize electrode placement to reduce PLE incidence.

Previous studies have indicated that older patient age is a significant risk factor for PLE [8]. Additionally, while longer operative time and increased microelectrode recordings (MERs) per electrode showed a trend towards higher risk, these factors did not reach statistical significance [8]. This suggests that the physical stress and duration of the procedure may contribute to developing edema, although further research is needed to confirm these associations. Additionally, we would expect there to be a significant association when comparing our StarFix group and robot-assisted group if this were the case.

Another study found that cortex volume and total gray matter volume were significantly correlated with the occurrence of PLE [10]. Specifically, lower volumes were associated with a higher incidence of PLE. This indicates that patients with greater brain atrophy, particularly in the gray matter, may be more susceptible to developing PLE post-DBS. Further research on brain volumes and the incidence of PLE would benefit our understanding of this phenomenon.

The potential mechanisms underlying these risk factors can be understood through the pathophysiology of PLE. Older age may be associated with reduced neuroplasticity and a diminished capacity for the brain to manage surgical trauma, leading to an increased risk of edema [8]. Longer operative times and more MERs could result in greater tissue manipulation and microvascular injury, contributing to fluid accumulation around the lead [8]. Reduced cortex and gray matter volumes may reflect underlying neurodegenerative changes that compromise the brain’s structural integrity and its ability to respond to surgical stress, thereby predisposing these patients to PLE [8, 10, 11].

### Differences between neural electrodes

Current efforts to minimize brain tissue damage and the subsequent inflammatory response to implanted neural electrodes have focused on several strategies, including modifying the electrode coating, utilizing biocompatible materials, and incorporating optimized geometries during the design process [12–15]. Interestingly, Boston Scientific is known to use many of these strategies in addition to further design changes to produce a more biocompatible electrode with a higher degree of stable placement [13, 16, 17].

Boston Scientific hardware differs from its competitors by employing microtextured surfaces to enhance tissue integration and reduce lead migration. This has been shown to minimize tissue damage and inflammatory responses, including peri-lead edema, particularly in older patients [13, 17]. However, all the major manufacturers utilize platinum-iridium alloy, an electrode material known for its biocompatibility and durability [17].

Although there are only subtle differences among competitors’ designs, the biggest differences are apparent in their stimulation technology and proposed mechanisms of action. Abbott’s neural electrodes have a feature known as BurstDR waveform, which is designed to generate signals at lower thresholds and with greater energy efficiency. This has been demonstrated by the production of EMG responses at significantly lower amplitudes than traditional tonic stimulation, indicating a more efficient and potentially less energy-consuming design [18]. Unlike Abbott’s BurstDR, Medtronic’s neural electrodes are characterized by their high-dose stimulation programming, which does not generate observable EMG response, suggesting a different mechanism of action [18]. Boston Scientific, on the other hand, aims to mimic the natural firing patterns of neurons by producing separate spikes consistent with traditional tonic stimulation without propagation [18]. It is possible that the varying mechanisms of action between products might contribute to the observed levels of PLE despite utilizing biocompatible materials and optimized geometries.

Recent advancements to counter complications with neural electrode implantation include silica nanoparticle (SiNP) coatings developed to enhance neural probe electrochemical properties and promote device-tissue integration [12]. These specialized coatings improve neuronal cell attachment and inhibit microglia activation, reducing inflammation and glial scarring. Additionally, conductive hydrogel coatings have provided a lower impedance and a higher charge injection limit, supporting the safe application of smaller electrode sizes [13]. Conductive hydrogel coatings typically comprise materials like PEDOT: PSS, which provide electrical conductivity and mechanical compliance, crucial for stable chronic neuromodulation [13]. While research into SiNP and conductive hydrogel coatings shows promise for improving the biocompatibility and performance of neural electrodes, leading manufacturers have not yet adopted these technologies for their commercially available DBS systems.

### Implications for patient care

The findings of this study highlight PLE as a relatively common but underappreciated complication following DBS surgery, with an incidence rate of nearly 49% among implanted electrodes. These results underscore the importance of heightened postoperative monitoring to detect PLE, particularly in cases involving Boston Scientific electrodes, which demonstrated a statistically significant association with this complication. These findings could influence the selection of electrodes for specific patient populations, particularly those with known risk factors or comorbidities that may predispose them to complications. While further studies are needed to confirm the relationship between specific lead brands and PLE, this study’s results encourage interdisciplinary collaboration between neurosurgeons and device manufacturers to optimize electrode design and reduce the risk of adverse outcomes. Incorporating these insights into clinical practice, healthcare providers can enhance the safety and effectiveness of DBS therapy, ultimately improving patient outcomes and satisfaction.

### Limitations

The study’s limitations include its retrospective design, which is inherently subject to selection bias, incomplete data, and limited control over confounding variables. While the findings provide valuable insights into PLE, the relatively small sample size may affect the statistical power and generalizability of the results, particularly for subgroup analyses, such as comparisons across electrode brands or surgical techniques. Additionally, this study only included patients treated by a single surgeon at a single institution, which may limit the applicability of the findings to broader, more diverse populations.

The reliance on postoperative CT imaging to assess edema volume, while practical, could have missed subtler forms of PLE that might be better detected with more sensitive modalities such as MRI. Moreover, the lack of detailed longitudinal follow-up data limits the ability to assess the long-term clinical impact of PLE, including its resolution or potential influence on device function and patient outcomes.

A significant limitation of the current study is the uneven distribution of patients using Abbott electrodes (*n* = 106) compared to Boston Scientific electrodes (*n* = 22). This imbalance could have contributed to the observed association between Boston Scientific electrodes and PLE. Since the primary outcome was not specifically powered for a comparison between these two groups, the effect observed may be due to this unequal distribution rather than a true difference between the electrode brands. More studies are needed that directly compare an equal number of patients using Abbott electrodes vs. Boston Scientific electrodes to draw more definitive conclusions regarding this potential relationship.

While a significant association between Boston Scientific electrodes and PLE was observed, causation cannot be established based on this study alone. Other factors—such as surgical technique, individual anatomical variations, or unmeasured confounders—may have influenced this outcome. Larger, multicenter, prospective studies are needed to overcome these limitations, clarify the underlying mechanisms of PLE, and determine whether modifications in electrode design or surgical protocols could reduce its occurrence. Additionally, future prospective research examining the correlation between physical symptoms and radiographic edema will be essential to better understand the clinical relevance of this phenomenon. The retrospective nature of the current study limited our ability to explore these relationships in depth.

## Conclusion

In conclusion, PLE is a well-recognized postoperative complication of DBS, typically presenting with delayed onset approximately two week after surgery. This study identifies a potential association between the use of Boston Scientific electrodes and an increased risk of PLE, suggesting that device-specific factors may play a role in its development, however, there are limitations to this study that need to be addressed prospectively. Furthermore, no significant associations were found between patient demographics or surgical technique. These findings underscore the importance of further prospective research to validate the observed association, explore the underlying mechanisms, and identify strategies to reduce PLE risk.

## Data Availability

No datasets were generated or analysed during the current study.
